# Smoothened is a poor prognosis factor and a potential therapeutic target in glioma

**DOI:** 10.1038/srep42630

**Published:** 2017-02-14

**Authors:** Yiming Tu, Mingshan Niu, Peng Xie, Chenglong Yue, Ning Liu, Zhenglei Qi, Shangfeng Gao, Hongmei Liu, Qiong Shi, Rutong Yu, Xuejiao Liu

**Affiliations:** 1 Insititute of Nervous System Diseases, Xuzhou Medical University, Xuzhou, Jiangsu, China; 2 Jiangsu Key Laboratory of Bone Marrow Stem Cell, Blood Diseases Institute, Xuzhou, Medical University, Xuzhou, Jiangsu, China; 3 Department of Neurosurgery, Huai’an Hospital Affiliated of Xuzhou Medical University and Huai’an Second People’s Hospital, Huaian, Jiangsu, China; 4 Brain Hospital, the Affiliated Hospital of Xuzhou Medical University, Xuzhou, Jiangsu, China

## Abstract

Malignant gliomas are associated with a high mortality rate. Thus, there is an urgent need for the development of novel targeted therapeutics. Aberrant Hedgehog signaling has been directly linked to glioma. GDC-0449 is a novel small molecule inhibitor of Hedgehog signaling that blocks the activity of smoothened (Smo). In this study, we evaluated the *in vitro* and *in vivo* effects of the smoothened inhibitor GDC-0449 on cell proliferation in human gliomas. We found that high expression of *smoothened* in glioma is a predictor of short overall survival and poor patient outcome. Our data suggest that GDC-0449 significantly inhibits the proliferation of glioma cells by inducing cell cycle arrest at the G1 phase. Our results demonstrate that GDC-0449 can effectively inhibit the migration and invasion of glioma cells. Furthermore, GDC-0449 treatment significantly suppressed glioma cell xenograft tumorigenesis. Mechanistically, GDC-0449 treatment markedly decreases the expression levels of key Hedgehog pathway component genes (*Shh, Patched-1, Patched-2, smoothened, Gli1* and *Gli2*). These results indicate that GDC-0449 works through targeting the Hedgehog pathway. Taken together, our study suggests that smoothened could be used as a prognostic marker and molecular therapeutic target for glioma.

Glioma is the most common form of malignant brain cancer in humans^1^. The standard treatment for glioma is surgery followed by external radiation and chemotherapy. However, the treatment is not optimal because of the high invasiveness, rapid progression and resistance to radiotherapy and chemotherapy^2^. Thus, there is an urgent need for the development of novel targeted therapeutics.

The Hedgehog pathway is a complex signaling cascade that performs crucial functions in cell proliferation, apoptosis and self-renewal^3^. Although this pathway is inactive in normal human mature cells^4^, it is hyper-activated in many tumors^5^. Deregulation of Hedgehog signaling has been implicated in the pathogenesis of glioma. Hedgehog signaling is initiated by Sonic Hedgehog (Shh) binding to its receptor^6^, patched, which thereby diminishes the inhibitory effects of patched-1 (PTCH1) on smoothened (Smo). Smoothened is then localized into the primary cilium of the cell, which plays a critical role in Hedgehog signaling^7^. Next, smoothened activates an intracellular cascade that leads to the activation and nuclear translocation of transcription factor Gli2^8^. Gli2 translocates into the nucleus and induces the transcription of Hedgehog target genes, such as Gli1^9^. Gli2 is a key component of Hedgehog signaling and its inactivation induces an inhibition of Hedgehog signaling^10^. Recent evidence shows that the Hedgehog signaling pathway at the level of Gli genes has an important role in glioma development^11^.

Targeting the Hedgehog pathway could be a promising therapy for gliomas. GDC-0449 (Vismodegib) is an inhibitor of the Hedgehog pathway that binds to smoothened^12^, which leads to inhibition of hyper-activated Hedgehog pathway^13^. Smoothened is the key site of Hedgehog pathway^14^. GDC-0449 has been identified in a cell-based small molecule screen for Gli family-mediated transcription inhibitor^15^. GDC-0449 acts as a potent inhibitor of smoothened and shows a high degree of selectivity for Hedgehog signaling. Preclinical data has demonstrated the antitumor activity of GDC-0449 in many human solid and hematologic malignancies^16^,^17^,^18^. In addition, GDC-0449 has entered clinical trial for glioma^19^. However, its underlying action mechanisms have not been investigated in glioma.

In the present study, we examined the association of smoothened expression with survival rate of glioma. Furthermore, we investigated the therapeutic efficacy of GDC-0449 against glioma *in vitro* and *in vivo*. These results suggested that GDC-0449 may be considered as a potential anticancer agent for therapy of gliomas through Hh pathway. Our finding support the development of GDC-0449 for the clinical treatment of glioma.

## Results

### GDC-0449 inhibits proliferation of glioma cells

**Figure 1:**
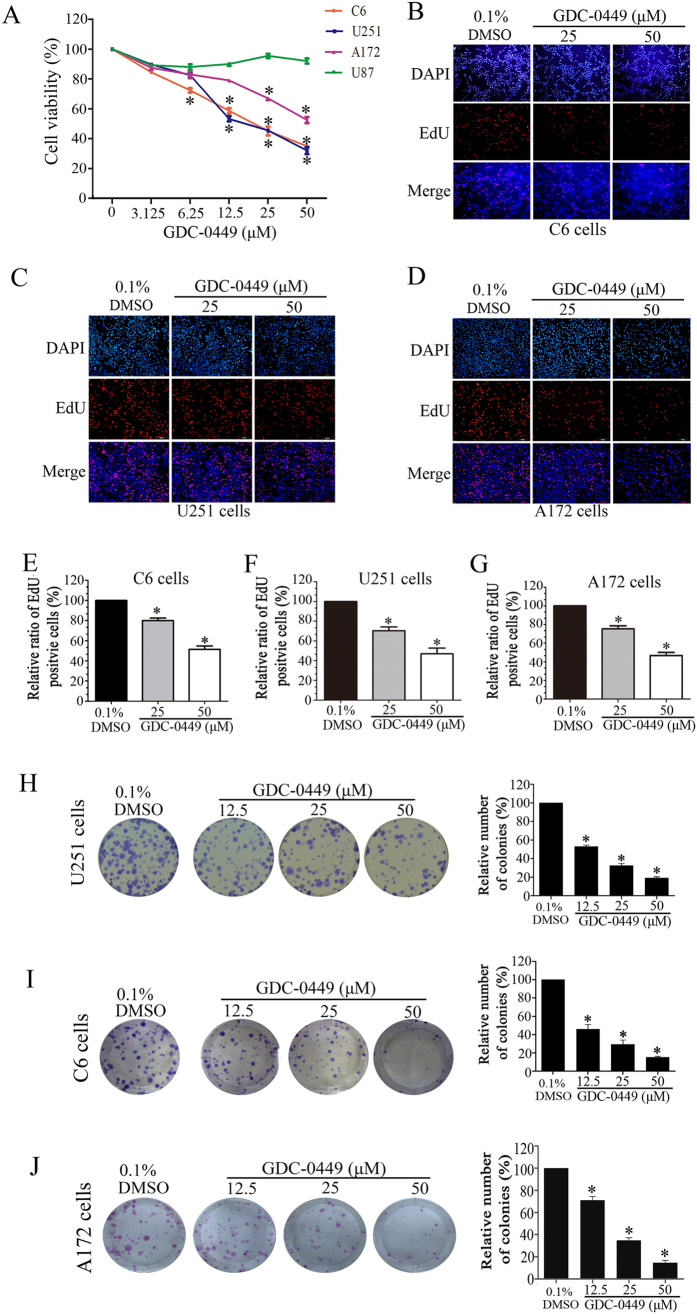
GDC-0449 inhibits proliferation of glioma cells. (**A**) Glioma cells were treated with the indicated concentrations of GDC-0449 for 72 h. Cell viability was measured by CCK-8 assay. (**B**–**D**) Representative images from EdU analysis of cell proliferation after C6, U251 and A172 cells were treated GDC-0449 at the indicated concentrations. (**E**–**G**) Quantitative results of EdU incorporation assay in C6, U251 and A172 cells. The percentage of proliferative cells was normalized to the corresponding values of the control group. Data were expressed as the means ± SEM from 3 independent experiments. (**H**–**J**) GDC-0449 inhibits the colony formation of U251, C6 and A172 cells, and quantitative results of clonogenic assay. The numbers of colony formation were normalized to the control group.

To assess the effect of GDC-0449 on the growth inhibition of glioma cells, four glioma cell lines were treated with GDC-0449 and cell viability was estimated using CCK8 assay. As shown in Fig. 1A, GDC-0449 significantly inhibited glioma cell proliferation in a dose-dependent manner in U251, C6 and A172 cells. The estimated IC_50_ values ranged from 12.5 to 25 μM in U251 or C6 cells. To confirm the anti-proliferative activity of GDC-0449, we tested the cell proliferation by EdU fluorescence staining assay. As shown in Fig. 1B–G, GDC-0449 treatment resulted in a significant reduction of the mean percentage of proliferating cells compared with the control group in C6, U251 and U87 cells. U251 cells exposed to 25 and 50 μM GDC-0449 reduced the proliferation to approximately 70.42% and 46.70%, respectively.

To evaluate the long-term effects of GDC-0449 on cell proliferation, a clonogenic formation assay was performed. As shown in Fig. 1H–J, GDC-0449 treatment induced a dose-dependent inhibition of the clonogenic potential of U251, C6 and A172 cells. Compared with the control group, the numbers of colony formation were markedly decreased to 53.11%, 32.76% and 19.09% in response to 12.5, 25 and 50 μM GDC-0449 treatment in U251 cells, respectively. Taken together, these results suggest that GDC-0449 can significantly inhibit the viability of glioma cells.

### GDC-0449 inhibits the migration and invasion of glioma cells

**Figure 2:**
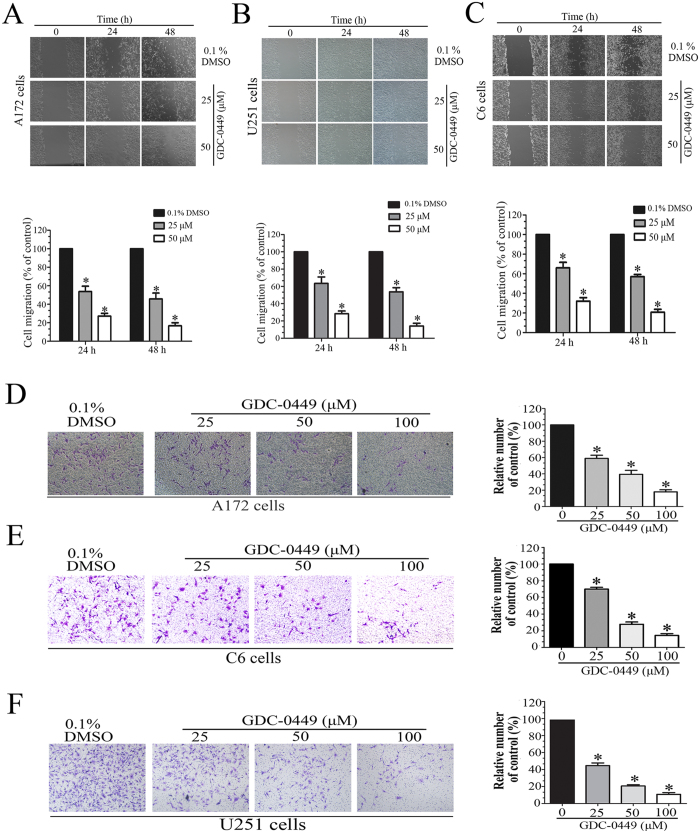
GDC-0449 inhibits the migration and invasion of glioma cells. (**A**–**C**) Effects of GDC-0449 on migration of A172, U251 and C6 cells as examined by wound healing assay. (**D**–**F**) Effects of GDC-0449 on invasion ability of A172, C6 and U251 cells as examined by transwell assay. The numbers of migratory or invading cells were normalized to the control group. The results are expressed as the mean ± SEM from three independent experiments.

We detected whether GDC-0449 had inhibitory effects on migration and invasion of glioma cells using *in vitro* wound healing and invasion assays. GDC-0449 significantly inhibited cell migration in a dose-dependent manner in A172, U251 and C6 cells (Fig. 2A–C). We found that 24 hours after being scratched, the migratory cell numbers of GDC-0449 treatment group were reduced to 45.80% and 16.7% in response to 25 and 50 μM GDC-0449 treatment, respectively, in A172 cells compared with control group. In addition to migration, the invasion assay showed that GDC-0449 induced a dose-dependent reduction of invasive cell numbers with increasing concentration of GDC-0449 (Fig. 2D–F). Compared with control group, the invasive cell numbers were reduced to 59.14%, 39.54% and 18.09% in response to 25, 50 and 100 μM GDC-0449 treatment in A172 cells, respectively (Fig. 2D). These results demonstrate that GDC-0449 can effectively inhibit the migration and invasion of glioma cells.

### GDC-0449 induces G1 arrest and modulates cell cycle regulators expression

**Figure 3:**
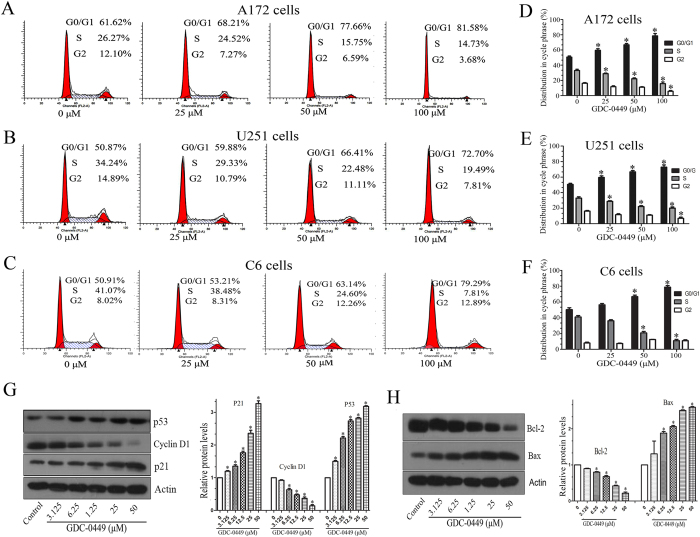
GDC-0449 induces cell cycle arrest in glioma cells. (**A**–**C**) Representative data from of the cell cycle analysis of GDC-0449-treated cells. A172, U251 and C6 cells were treated with GDC-0449 at the indicated concentrations for 24 h. Cells were stained with PI and evaluated using flow cytometry. (**D**–**F**) Quantitative analysis of cycle phase distribution in the control group and the GC-0449-treated group. (**G**,**H**) GDC-0449 treatment affected the expression levels of cell cycle-related protein levels. U251 cells were treated with 0.1% DMSO or GDC-0449 at the indicated concentrations for 24 h. Cells were then harvested and examined using Western blot analysis with the indicated antibodies. Quantitative results of Western blot assay are expressed as the mean ± SEM from three independent experiments.

To investigate whether the GDC-0449-induced decrease in cell proliferation resulted from the abrogation of cell cycle progression, we evaluated the cell cycle distribution using flow cytometry assay. As shown in Fig. 3A–F, the U251, A172 and C6 cells were arrested at G1 phase of the cell cycle in response to treatment with GDC-0449. In A172 cells treated with the DMSO vehicle, 61.2% of cells were in the G1 fraction, whereas cells treated with 50 and 100 μM GDC-0449 exhibited a higher population of cells (77.6% and 81.6%, respectively) in the G1 phase (Fig. 3A). In addition, a significant decrease in the S phase populations compared with the control group was also observed.

We next examined whether GDC-0449 modulates cell cycle regulatory proteins to induce G1 arrest using Western blot analysis. GDC-0449 treatment significantly enhanced the expression levels of cell cycle inhibitory proteins p27, p53 and Bax in a dose-dependent manner (Fig. 3G). In addition, the expression levels of Cyclin D1 and Bcl-2 were significantly reduced in GDC-0449-treated cells compared with control cells (Fig. 3H). These results suggest that GDC-0449 induces G1 arrest in glioma cells by modulating multiple cell cycle regulatory proteins.

### High smoothened expression predicts poor survival in patients with glioma

**Figure 4:**
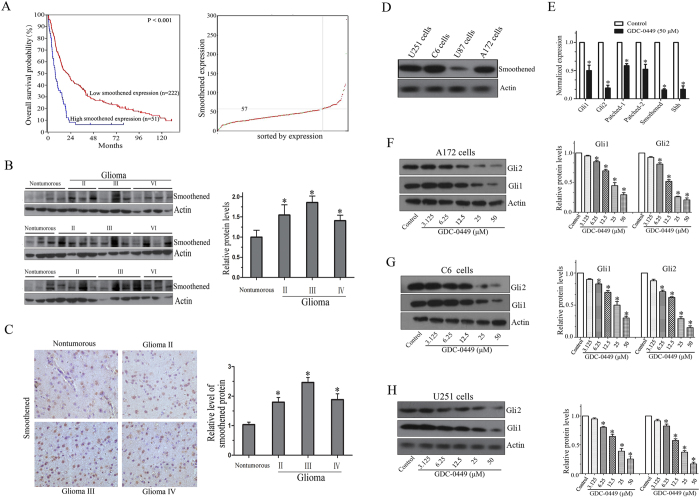
GDC-0449 suppresses Hedgehog pathway in glioma cells. (**A**) Kaplan-Meier analysis of overall survival for the French data. Smoothened had high expression in 51 out of 273 cases of glioma was associated with poor patient survival. (**B**) Total protein extracts isolated from non-tumorous brain tissues and glioma tissues were evaluated using Western blotting assays with the anti-smoothened antibodies. Quantitative results of Western blot assay are expressed as the mean ± SEM. (**C**) Immunohistochemistry staining of non-tumorous brain tissues and glioma tissues for the expression of smoothened. Quantitative analyses of the percentages of positive cells in the glioma group normalized to the non-tumorous group. These results are presented as the means ± SEM of four independent experiments. (**D**) Total protein extracts isolated from four glioma cell lines were evaluated using Western blotting assays with the anti-smoothened antibodies. (**E**) A172 cells were treated with GDC-0449 for 24 h. The mRNA expression of Hedgehog pathway gene examined using real time RT-PCR. (**F**–**H**) A172, C6 and U251 cells were treated with 0.1% DMSO or GDC-0449 for 24 h. Gli1 and Gli2 protein levels were examined by Western blot analysis. Quantitative results of Western blot assay are expressed as the means ± SEM from three independent experiments.

To evaluate the possibility that smoothened is important for glioma, we analyzed the R2 genomics database, for which microarray-based gene expression and clinical outcome data were available. The prognosis analysis was conducted online and cutoff values for separating high and low expression groups were determine by auto scan. As shown in Fig. 4A, *smoothened* gene was highly expressed in 51 out of 273 cases of glioma. The distinction between high and low *smoothened* was of prognostic significance, as the overall survival rate was markedly reduced in cases exhibiting high *smoothened* expression. Next, we assessed smoothened protein expression in human glioma tissues through a Western blot and immunohistochemistry staining analysis and found that smoothened was highly expressed in tumor samples compared with non-tumorous brain tissues (Fig. 4B and C). Furthermore, smoothened protein was highly expressed in U251, C6 and A172 cells, but a low expression was found in U87 cells (Fig. 4D). These findings indicate that up-regulation of *smoothened* in a subset of glioma leads to an inferior outcome.

### GDC-0449 targets Hedgehog pathway in glioma cells

To further understand the molecular mechanism involved in GDC-0449-induced cell growth inhibition, alterations in the Hedgehog pathway were investigated using real-time RT-PCR and Western blotting analysis. As shown in Fig. 4F–H, GDC-0449 treatment leads to significant down-regulation of Gli1and Gli2 protein compared to control. Furthermore, A172 cells treated with GDC-0449 were assessed using real-time RT-PCR. As shown in Fig. 4E, the mRNA expression of Hedgehog pathway genes (*Shh, Patched-1, Patched-2, smoothened, Gli1* and *Gli2*) were all significantly decreased after GDC-0449 treatment. These results suggest that GDC-0449 works through suppressing the Hedgehog pathway.

### GDC-0449 suppresses glioma xenograft tumorigenesis *in vivo*

**Figure 5:**
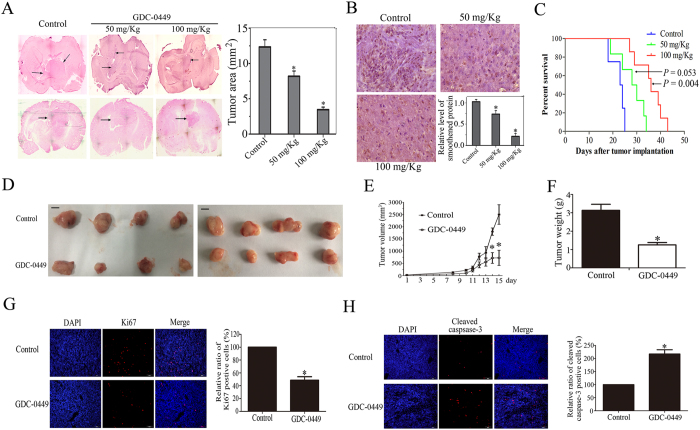
GDC-0449 inhibits glioma tumorigenesis *in vivo*. (**A**) C6 cells (1 × 10^5^ cells per mouse) were intracranially injected into the right striatum of BALB/c nude mice using a small animal stereotactic apparatus. Representative images of H&E staining of coronal sections from mouse brains with orthotopic tumors. The mean tumor areas were measured. The data are expressed as the means ± SEM (n = 3). (**B**) Immunohistochemistry staining of xenograft tumor tissues for the expression of smoothened. Quantitative analyses of the percentages of positive cells in the GDC-0449-treated group normalized to the control group. These results are presented as the means ± SEM of three independent experiments. (**C**) The survival of mice with intracranial tumors derived from the vehicle or GDC0449-treated groups was measured by Kaplan-Meier survival curves. (**D**) C6 cells were pretreated with DMSO or GDC-0449 and subcutaneously injected into nude mice. After 5 days of tumor injection, the tumor-bearing mice were randomly divided into one of the following three treatment groups (n = 10 per group). After 15 days, mice were sacrificed and tumors were collected to assay the tumor diameter and weight. Representative tumors isolated from the control and GDC-0449-treated groups. Scale bar: 10 mm. (**E**) The mean tumor volumes were assessed at the indicated days after GDC-0449 treatment. (**F**) The mean tumor weights were measured. The data are expressed as the means ± SEM. (**G**,**H**) Cell proliferation and apoptosis of the subcutaneous tumor were assessed with anti-Ki67 and anti-cleaved caspase-3 immunostaining. Quantitative analyses of the percentages of positive cells in the GDC-0449-treated group normalized to the control group. These results are presented as the means ± SEM of three independent experiments.

To determine whether GDC-0449 exerts anti-tumor activity on glioma cells *in vivo*, we evaluated its effect in an intracranial nude mouse model. As shown in Fig. 5A, 15 days after glioma transplantation, the transplanted gliomas in GDC-0449-treated mice were visibly smaller than those in the vehicle-treated mice. Immunohistochemistry staining analysis of xenograft tumors also revealed that GDC-0449 treatment reduced the expression of smoothened compared with the control group (Fig. 5B). Consequently, survival analysis of GDC-0449 demonstrated significant benefit in GDC-0449-treated mice compared to vehicle-treated mice (Fig. 5C).

We next carried out xenograft experiments by subdermal transplantation with donor glioma cells pretreated with DMSO vehicle or GDC-0449. As shown in Fig. 5D and E, GDC-0449 pretreatment significantly suppressed xenograft tumorigenesis and reduced the size of tumors. The mean weight of control group tumors was significantly higher compared with that of GDC-0449-treated tumors. These results demonstrate that GDC-0449 significantly inhibits glioma cells growth *in vivo*.

To further evaluate the effects of GDC-0449 on tumor growth, Ki67 and cleaved caspase-3 were used to show cell proliferation and apoptosis in xenografts, respectively^20^. The percentage of Ki67-positive cells in tumors was decreased by 41.5% in the GDC-0449-treated group (Fig. 5G). In contrast, the percentage of apoptotic cells was significantly increased by 117.25% in the GDC-0449 treated-group (Fig. 5H). These data suggest that GDC-0449 can inhibit tumor cell proliferation and induce cell apoptosis *in vivo*.

## Discussion

Glioma has the highest incidence and cancer-associated mortality rates of malignant brain cancers^21^. However, there have been no effective targeted treatment strategies for glioma until now. Therefore, new modalities that can improve current treatments for gliomas are highly desirable. In this study, GDC-0449 demonstrated antitumor effects in glioma models *in vitro* and *in vivo*. Furthermore, our data show that GDC-0449 exerts its anti-tumor effects by targeting Hedgehog pathway in glioma.

Hedgehog signaling has been involved in the initiation and maintenance of glioma^22^. Hedgehog signaling events have been implicated in tumor cell proliferation and survival as well as the molecular hallmark of different human tumors^23^. Smoothened is a transmembrane protein that activates the down-stream Hedgehog signaling pathway. However, to our best knowledge, there is little information about prognostic status and clinical outcome of smoothened expression in glioma. We found that high expression of *smoothened* in glioma is a predictor of short overall survival. These findings suggest that overexpression of *smoothened* in glioma may represent an acquired malignant phenotypic feature of tumor cells.

GDC-0449, an orally bioavailable, small molecule smoothened inhibitor, was recently approved for advanced basal cell carcinoma and is being evaluated in other tumors^23^,^24^. In this study, we demonstrated that GDC-0449 significantly suppressed proliferation in glioma cells both *in vitro* and *in vivo*. We also found that GDC-0449 could inhibit glioma cell migration and invasion at lower doses. The anti-proliferative activity of GDC-0449 results from the induction of cell cycle arrest in the G1 phase. Interestingly, we found the U87 cell line was not responsive towards smoothened inhibitor GDC-0449 (Fig. 1A). It may be caused by the possible low expression level of smoothened in U87 cells (Fig. 4C). Identifying the mechanisms of acquired resistance to selective Hedgehog pathway inhibitors in patents with glioma will be of particular interest in future studies.

Aberrant smoothened signaling activation has been associated with cancer progression^25^. Smoothened promotes the translocation of Gli transcription factors into the nucleus to bind to their target genes, including Gli1, Patched-1, Myc and Bcl2^26^. We found that GDC-0449 treatment significantly decreased the expression levels of Gli1 and Gli2 in glioma cells. Gli genes are members of Hedgehog pathway, which act as downstream mediators of Hedgehog signaling and they have regulatory effects on cell cycle^26^. In the present study, exposure to GDC-0449 induced significant cell cycle arrest in human glioma cells. Interestingly, GDC-0449 treatment markedly inhibited expressions of Hedgehog receptors, including *Patched-1, Patched-2* and *smoothened*. Furthermore, Bcl-2 was down-regulated following the treatment of GDC-0449 in glioma cells. Bcl-2 is a key anti-apoptotic protein in Hedgehog-dependent cell survival^27^. Our results highlight the critical role of Hedgehog signaling in human glioma cells.

In summary, our data indicate that smoothened could be used as a prognostic molecular marker and therapeutic target for glioma. Our results presented experimental evidence which strongly supports that GDC-0449 may function as a potential natural inhibitor of Hedgehog pathway, ultimately causing cell growth inhibition and cell cycle arrest. In particular, our results suggest that the underlying mechanism of action of GDC-0449 involves suppressing the Hedgehog pathway. These findings highlight the potential of future clinical trials evaluating the therapeutic potential of GDC-0449 for the treatment of human gliomas.

## Materials and Methods

### Cell culture and reagents

Human glioma cell lines U87, A172, U251 and mice glioma cell line C6 were cultured in DMEM supplemented with 10% FBS. These cell lines were grown in a humidified incubator containing 5% CO_2_ at 37 °C. Primary antibodies against Gli1, Gli2, Cyclin D1, p53, p21, Bcl-2, Bax and b-actin were obtained from Cell Signaling Technology.

#### Glioma and non-tumor human brain tissues

Human glioma specimens (obtained from surgical resection) and non-tumorous brain tissues (obtained from patients with internal decompression in cerebral trauma) were obtained from Affiliated Hospital of Xuzhou Medical University (Xuzhou, China). All of the patient tissue samples used in this study were immediately frozen in liquid nitrogen after surgical resection and stored at −80 °C. Written informed consent was obtained from all participants and this study was approved by the ethics committee of the Affiliated Hospital of Xuzhou Medical University. All experiments were performed in accordance with the relevant guidelines and regulations of Affiliated Hospital of Xuzhou Medical University.

### Cell viability assay

Cell proliferation was assessed with the Cell Counting Kit-8 (CCK8) assay. The glioma cells (3 × 10^3^) were seeded on 96-well plates and cultured overnight to allow the cells to attach to the plates. The cells were treated with various concentrations of GDC-0449 (3.125, 6.25, 12.5, 25 and 50 μM) for 72 h. Ten microliters of CCK8 was added to each well. Following a 3 h incubation, the absorbance was measured at 450 nm using a spectrophotometer^20^.

### EdU assay

Cell proliferation was assessed by 5-ethynyl-20-deoxyuridine (EdU) fluorescence staining using the Cell-Light TM EdU DNA cell proliferation kit (Ruibo Biotech, Guangzhou, China). The A172, U251 and C6 cells were seeded in 96-well culture plates and incubated overnight, respectively. Then, the cells were treated with 0.1% DMSO (vehicle) or GDC-0449 (25 and 50 μM) for 12 h. Subsequently, cells were incubated with 50 μM EdU for 4 h. Cells were then fixed with 4% paraformaldehyde for 15 min and treated with 0.5% Triton X-100 for 20 min. Next, the cells were incubated with 100 μL of 1 × Apollo® reaction cocktail for 30 min and stained with DAPI for 15 min. After washing with phosphate-buffered saline (PBS) for 3 times, the cells were examined with fluorescence microscopy and photographed (Olympus, Japan)^28^.

### Cell cycle analysis

The effect of GDC-0449 treatment on cell cycle distribution was detected by using flow cytometry analysis. Briefly, A172 and U251 cells were treated with 25, 50, 100 μM GDC0449 for 24 h, respectively. After treatment, cells were collected and fixed in 70% ethanol. The cells were then washed with PBS for two times stained with PI solution that contained 50 μg/mL PI and 25 μg/mL RNAse for 30 min. Finally, cells were assayed on a FACSCalibur (Becton-Dickinson) and analyzed by CellQuest Pro software (BectonDickinson).

### Wound migration assay

The migration behavior of glioma cells was evaluated by using wound healing assay. Briefly, glioma cells were seeded in 24-well plates and allowed to attach overnight. A rectangular lesion was created using a plastic pipette tip and incubated in serum-free media. Cells were treated with either 0.1% DMSO or GDC-0449. After 24 h and 48 h incubation, five randomly selected fields at the lesion border were acquired under microscope.

### Transwell invasion assays

Cell invasion assays was performed using a transwell system. Culture inserts were coated with matrigel and placed into the wells of 24-well culture plates. Cells treated with either 0.1% DMSO or GDC-0449 in serum-free media were added to the top chamber. In the lower chamber, the DMEM media containing 10% FBS was added. After 36 h of incubation, the media was removed and the cells were fixed in 4% methanol for 20 min and stained with a 0.3% crystal violet solution for 30 min. The observation of the migratory cells was recorded with microscopy.

### Western blotting

Cells were treated with different concentrations of GDC-0449 (3.125, 6.25, 12.5, 25 and 50 μM). After 24 h of incubation, total protein extracts from treated cells were subjected to Western blot analysis as described previously^29^. The expression patterns of Gli1, Gli2, cyclin D1, p53, p21, Bcl-2, Bax were detected using specific antibodies and actin was used as the loading control. Each experiment was carried out in three biological replicates and average fold changes are reported.

### Gene expression analysis

Total RNA was isolated using TRIzol (Invitrogen) according to the manufacturer’s protocol. Briefly, RNA was isolated and reverse transcribed cDNA using cDNA Reverse transcription kit (Roche). The following gene-specific primers were used: smoothened (5′-TCGCTACCCTGCTGTTATTC-3′, 5′-GACGCAGGACAGAGTCTCAT-3′), Patched1 (5′-TGACCTAGTCAGGCTGGA AG-3′, 5′-GAAGGAGATTATCCCCCTGA-3′), Patched2 (5′-AGGAGCTGCATTACACCAAG-3′, 5′-CCCAGGACTTCCCATAGAGT-3′), Gli1 (5′-CTGGATCGGATAGGTGGTCT-3′, 5′-CAGAGGTTGGGAGGTAAGGA-3′), Gli2 (5′-GCCCTTCCTGAAAAGAAGAC-3′, 5′- CATTGGAGAAACAGGATTGG-3′), GAPDH (5′-GAGTCAACGGATTTGGTCGT-3′, 5′-TTGATTTTGGAGGGATCTCG-3′). The applied PCR conditions were 50 °C for 2 min and 95 °C for 3 min followed by 40 cycles at 95 °C for 15 sec and at 60 °C for 1 min. Data were acquired and processed automatically by the Applied Biosystems 7500 Real-Time PCR System.

#### Intracranial orthotopic glioma model

All animal experimental protocols were approved by the Ethics Committee of the Xuzhou Medical University. All experiments were performed in accordance with the relevant guidelines and regulations of Xuzhou Medical University. Male athymic BALB/c nude mice aged five to six weeks were obtained from the Experimental Animal Center of Xuzhou Medical University. C6 cells (1 × 10^5^ cells per mouse) were intracranially injected into the right striatum of nude mice using a small animal stereotactic apparatus. After 5 days of tumor injection, the tumor-bearing mice were randomly divided into one of the following three treatment groups (n = 10 per group): GDC-0449 at 50 mg/kg, GDC-0449 at 100 mg/kg and vehicle. The drugs and vehicle were delivered daily via intraperitoneal injections. Three mice of each group were sacrificed 15 days after glioma transplantation. The tumors of the control and treated mice were harvested and prepared for histological study.

#### Histopathology

The whole brain of the control and treated mice were fixed in 4% paraformaldehyde and dehydrated sequentially in 20% and 30% sucrose at 4 °C until they sank. The frozen glioma tissues were serially sectioned at a thickness of 12 μm and the slide with the largest tumor area was stained with hematoxylin and eosin (H&E). Images were acquired using a microscope with a fluorescence detector attached (IX71 Olympus).

### Tumor xenograft study

In experiments, C6 cells were pretreated with GDC-0449 (50 μM) or 0.1% DMSO for 48 hours. Cells were then harvested and resuspended in L15 with GDC-0449 (50 μM) or 0.1% DMSO. The cell number was counted approach approximately 1.0 × 10^7^/mL. Two hundred microliters of cell suspension was injected into male BALB/c immunocompromised mice (6 weeks old) bilaterally (GDC-0449 treatment cells into the right side and 0.1% DMSO control into the left side). Tumor growth was monitored daily by caliper measurement. After 15 days of injection, mice were sacrificed and the tumors were photographed and prepared for immunofluorescence analysis.

#### Immunofluorescence staining

The subcutaneous tumor of the control and treated mice were fixed in 4% paraformaldehyde and dehydrated sequentially in 20% and 30% sucrose at 4 °C until they sank. The frozen tumor tissues were serially sectioned at a thickness. The sections containing the tumor were incubated with 0.3% triton X-100 followed by 10% goat serum. Next, the tumors were incubated overnight with Ki67 or cleaved caspase-3 primary antibody. To visualize the Ki67-positive or cleaved caspase-3-positive cells, the sections were incubated with Alexa-594-conjugated secondary antibody for 1 h at room temperature in the dark. DAPI was used to stain the cell nuclei. All sections were examined and photographed with a microscope with an attached fluorescence detector (IX71 Olympus).

#### Smoothened expression and survival analysis in patients with glioma

Smoothened gene expression datasets were obtained from R2: microarray analysis and visualization platform (http://r2.amc.nl). We used the publicly available data of the R2 French glioma cohort. These glioma samples were collected from the tumor archive of the Erasmus University Medical Center, Rotterdam, Netherlands. All histologic diagnoses were made on formalin-fixed, paraffin-embedded hematoxylin and eosin stained sections and were reviewed blinded to the original diagnosis according to the 2007 WHO classification. The Kaplan-Meier analysis was conducted online and cutoff values for separating high and low expression groups were determine by Kaplan scanner. The Kaplan scanner will try to separate the gene expression data for a gene into 2 groups and determine the best possible Kaplan Meier curve.

### Statistical analysis

The results are presented as the means ± SEM of three to five independent experiments. Comparisons between untreated group and GDC-0449 treated group were calculated using Student’s *t* test. *P* values < 0.05 were considered statistically significant.

## Additional Information

**How to cite this article**: Tu, Y. *et al*. Smoothened is a poor prognosis factor and a potential therapeutic target in glioma. *Sci. Rep.* **7**, 42630; doi: 10.1038/srep42630 (2017).

**Publisher's note:** Springer Nature remains neutral with regard to jurisdictional claims in published maps and institutional affiliations.
